# Discovery of Genomic Characteristics and Selection Signatures in Southern Chinese Local Cattle

**DOI:** 10.3389/fgene.2020.533052

**Published:** 2020-12-18

**Authors:** Yuqiang Liu, Lingyang Xu, Liu Yang, Guoyao Zhao, Junya Li, Dewu Liu, Yaokun Li

**Affiliations:** ^1^College of Animal Science, South China Agricultural University, Guangzhou, China; ^2^Innovation Team of Cattle Genetic Breeding, Institute of Animal Science, Chinese Academy of Agricultural Sciences, Beijing, China; ^3^Lingnan Guangdong Laboratory of Modern Agriculture, Guangzhou, China; ^4^Farm Animal Genetic Resources Exploration and Innovation Key Laboratory of Sichuan Province, Sichuan Agricultural University, Chengdu, China

**Keywords:** genetic divergence, selection signature, population admixture, neighbor-joining tree, Chinese local cattle

## Abstract

Chinese local cattle with a high level of genetic diversity mainly originate from two subspecies; the cattle in northern China are primarily *Bos Taurus*, and the cattle in southern China are primarily *Bos indicus*. Cattle from southern China are characterized by a specific phenotype and adapted to the local environment. This study explored the genetic diversity, degree of admixture, and selection signature in eight local cattle breeds in southern China. The lowest level of heterozygosity was found in Hainan and Nandan cattle from Hainan and Guangxi province, respectively, whereas the highest level of heterozygosity was detected in Zhaotong cattle from Yunnan province. A neighbor-joining phylogenetic tree analysis clearly separated Lufeng cattle from other breeds, whereas Leiqiong and Hainan cattle have some crossover. Based on linkage disequilibrium-filtered single nucleotide polymorphisms (SNPs), the admixture analysis revealed two clusters corresponding to the taurine and indicine cattle lineages, and the local cattle breeds from southern China showed a certain degree of admixture. When *K* = 4 and 9, we found a slight separation among Leiqiong, Lufeng, and Hainan cattle. Meanwhile, we performed a selection signature analysis in Hainan, Leiqiong, and Lufeng cattle distributed in the extreme south of China, using the integrated haplotype score (iHS), Rsb statistic, and BayeScan software. Using the iHS approach, we identified 251, 270, and 256 candidate regions in Lufeng, Leiqiong, and Hainan cattle, respectively. Moreover, we identified 184, 174, and 146 candidate regions in pairwise comparisons of Leiqiong vs. Lufeng, Leiqiong vs. Hainan, and Hainan vs. Lufeng cattle using the Rsb approach. In addition, we identified 76 loci with a total of 48 genes under selection, based on the F_*ST*_ approach. Several candidate genes under selection were found to be related to meat quality, immunity, and adaptation to the local environment in southern China. Our results provide significant information about the genetic differences among the cattle breeds from southern China and the possible cause of difference in breed-specific characteristics. Selection signature analysis identified a few candidate SNPs and genes related to certain important traits of these cattle. In general, our results provide valuable insights into the genetic basis of specific traits under selection in certain local cattle breeds.

## Introduction

Cattle are important livestock species worldwide as humans obtain meat, milk, and leather from cattle. Most local cattle in China are classified into two categories based on physical appearance and genomic differences: *Bos taurus* cattle (humpless) and *B. indicus* cattle (humped) (Bradley et al., 1996; Lei et al., 2006; Zeder, 2008). The formation of different breeds and the selection history of the local environment provide a valuable genetic resource to investigate the genetic basis of complex traits (Bovine HapMap Consortium et al., 2009; Xu et al., 2019a).

In China, numerous cattle breeds are raised in diverse environmental conditions, and they can be divided into three groups based on the morphological characteristics and geographic distributions in China. The southern distributed breeds belong primarily to the subspecies *B. indicus*, which is resistant to damp heat and mites and exhibits a small but robust and compact constitution (Li et al., 2013). The northern distributed breeds belong primarily to the subspecies *B. taurus*, which has large body size, broad breast, and thick skin. The cattle breeds distributed centrally between the northern and southern regions are mostly *B. taurus* and *B. indicus* hybrids. Previous studies based on Y chromosome polymorphisms and mitochondrial DNA indicate a declining south-to-north gradient of indicine introgression (Cai et al., 2006; Lei et al., 2006; Xia et al., 2019).

Cattle raised in southern China are adaptive to hot and humid climates and have excellent meat quality and strong disease resistance. Natural selection and artificial selection together with introgression and genetic drift events could cause changes in the cattle genome. The distinct environment has also shaped the genomic changes in local cattle, which generate the selection signature across the bovine genome (Andersson and Georges, 2004). Therefore, it is important to investigate the population genetic structure, admixture, and selection signature of the cattle from southern China showing specific environmental characteristics. In recent studies, single nucleotide polymorphism (SNP) array and next-generation sequencing technologies are widely applied to analyze economically important and adaptive traits of livestock. Such studies have been performed to explore the population structure and selection signature in many animal species such as dog (Akey et al., 2010), pig (Gurgul et al., 2018), chicken (Yin et al., 2019), and horse (Petersen et al., 2013) cattle (Gao et al., 2017; Chen et al., 2018). The application of high-density SNP arrays can further improve the detection of positive selection and reduce the false discovery rate (O'Brien et al., 2014; Xu et al., 2015; Zhao et al., 2015; Gonzalez-Rodriguez et al., 2016).

The present study aimed to (1) investigate the population structure of the cattle breeds in southern China, (2) identify the selection signature associated with important traits of the cattle, and (3) pinpoint the potential candidate genes associated with adaptive traits in southern Chinese local cattle.

## Materials and Methods

### Ethics Approval Statement

The southern Chinese local cattle (LQC, LFC, and HNC) used in this study were owned by farmers. Before sampling, the objectives of the study and procedures involving animal sample collection were explained to the farmers, and they provided consent to collect samples from their animals. Animal welfare and health conditions were observed during the sampling process. All animal research protocols were approved by the Institutional Animal Care and Use Committees (IACUCs) of South China Agricultural University (Approval No. 2018-P002). Other animal genetic data used in this study were derived from previous analyses that have been specifically licensed (Xu et al., 2019a).

### Genotyping and Quality Control

A total of 201 individuals from eight cattle breeds were included in the study. The data on five of these breeds were obtained from a previous study, including Liangshan cattle (LSC; *n* = 22), Nandan cattle (NDC; *n* = 23), Pingwu cattle (PWC; *n* = 23), Wenshan cattle (WSC; *n* = 21), and Zhaotong cattle (ZTC; *n* = 23) (Xu et al., 2019a). The samples from the other three breeds were obtained from the extreme south of China, including Hainan cattle (HNC; *n* = 26), Leiqiong cattle (LQC; *n* = 30), and Lufeng cattle (LFC; *n* = 33). The breed name, abbreviation, and other information are presented in Table 1. All individuals were genotyped using the Illumina Bovine HD SNP array. We used PLINK v1.9 for SNP quality control and custom R scripts for data processing (Purcell et al., 2007). We pruned out individuals and loci based on the following criteria: (1) markers with >0.90 call rate; (2) minor allele frequency (MAF) of SNPs >0.05; (3) presence of SNPs only on autosomes; closely related individuals were excluded (PI-HAT value <0.25); (4) individual call rate >0.95.

**Table 1:** The genetic diversity analysis of the eight experimental cattle breeds.

**Breed**	**Abbreviation**	**Size**	**MAF**	**H_**o**_**	**H_**e**_**	**F-ROH**
Leiqiong	LQC	30	0.22	0.306	0.305	0.100
Lufeng	LFC	33	0.19	0.262	0.268	0.128
Hainan	HNC	26	0.19	0.254	0.261	0.153
Liangshan	LSC	22	0.28	0.372	0.366	0.041
Nandan	NDC	23	0.18	0.247	0.249	0.124
Pingwu	PWC	23	0.29	0.381	0.379	0.057
Wenshan	WSC	22	0.23	0.311	0.314	0.090
Zhaotong	ZTC	23	0.28	0.372	0.374	0.058

*MAF, Minor Allele Frequency; H_o_, Observed heterozygosity; H_e_, Expected heterozygosity; F-ROH, Inbreeding coefficient*.

### Genetic Diversity, Heterozygosity, and Effective Population Size

We performed a genetic diversity analysis for the eight cattle breeds from southern China. The observed heterozygosity (H_o_) and expected heterozygosity (H_e_) were estimated using PLINK v1.9 with the option “hardy.” We estimated the molecular inbreeding coefficient based on runs of homozygosity, as described previously (Purfield et al., 2012). SNeP software v1.1 was used to estimate the effective population size (*Ne*) (Barbato et al., 2017); the *Ne* value of the past t generations was inferred based on LD (Barbato et al., 2017).

### Population Structure and Admixture Analysis

After filtering for linkage disequilibrium (LD; using the parameter “–indep-pairwise 50 5 0.2”), a total of 62,486 SNPs were obtained for the multidimensional scaling analysis. Pairwise genome-wide identity-by-state (IBS) distances were estimated for sample clusters using PLINK v1.9 (-mds-plot 4). After strict LD-based filtering (*r*^2^ > 0.02), we extracted 11,455 SNPs to assess population admixture using STRUCTURE 2.3.4 (Pritchard et al., 2000; Falush et al., 2003). Each process was implemented using 10,000 replicates and 10,000 burn-in cycles under admixture and correlated allele frequency models. We estimated the genetic distance (D) between pairwise combinations of individuals using PLINK v1.9 (Purcell et al., 2007) using the formula D = 1–(IBS2 + 0.5 × IBS1)/*N*, where IBS1 and IBS2 are the number of loci that share either one or two alleles identical in state, respectively, and N is the total number of loci (Stevens et al., 2011). PHYLIP v3.69 software was used to estimate a neighbor-joining phylogenetic tree analysis; the phylogenetic tree was finally generated using Figtree 1.4.4 software, as reported previously (Yu et al., 2017). Finally, we used Treemix v1.13 to examine the history of splits and admixtures in the local cattle breeds of southern China (Pickrell and Pritchard, 2012). To further explore the evidence of admixture in the experimental cattle breeds, three-population (f3) and four-population (f4) tests implemented in Treemix v1.13 were utilized to examine the admixture (Reich et al., 2009; Pickrell and Pritchard, 2012).

### Identification of Selection Signatures

BayeScan v2.1 software was used to detect the selection signatures in LFC vs. LQC vs. HNC, LFC vs. LQC, LQC vs. HNC, and LFC vs. HNC, respectively (Foll and Gaggiotti, 2008). To avoid false positives, the prior probability of the model was set to 10 (using the default parameters in the BayeScan v2.1 software), the *q* < 0.01(de Simoni Gouveia et al., 2017), and F_*ST*_ > 0.15. The integrated haplotype score (iHS) was estimated using *selscan* with default settings (except for the maximum gap, which was set as 800,000) (Szpiech and Hernandez, 2014). The selection signals were detected by establishing a 100 kb non-overlapping window for the genomic regions. Single-site iHS values were computed across the genome in the experimental cattle breeds of southern China. The top 1% of regions with the highest average |iHS| scores and the windows with an SNP number >10 was used for subsequent analysis (Voight et al., 2006; Xu et al., 2017). The rehh package in R was used to estimate the Rsb value for the pairwise comparisons (Gautier and Vitalis, 2012). We used fastPHASE software to impute the missing genotype data; the parameters were set as follows: -H-4 -K10 -T10 -C25 (Scheet and Stephens, 2006). For Rsb estimation, the genotype data of the ancestors were obtained from a previous study (Utsunomiya et al., 2013). The Rsb analysis was estimated for each pairwise comparison among the three breeds LFC, LQC, and HNC. We explored the candidate genes located within the identified regions, which were defined by using the reference genome based on the UMD 3.1/bosTau6 assembly from the University of California Santa Cruz (UCSC) genome browser (Karolchik et al., 2004).

### Functional Analysis of the Candidate Genes

Gene ontology (GO) and Kyoto Encyclopedia of Genes and Genomes (KEGG) pathway enrichment analyses were estimated using Metascape with default parameters (Zhou et al., 2019; He et al., 2020).

## Results

### Genetic Diversity, Heterozygosity, and Current Effective Population Size

After quality control, 568,129 SNPs on autosomes were identified for downstream analysis. The MAF analysis revealed distinct patterns among different breeds. The average MAF in the eight cattle breeds of southern China was 0.18–0.29 (Table 1). Among the experimental cattle breeds, LFC and HNC had low MAF. In contrast, PWC had higher MAF. The genetic diversity across breeds was further investigated by estimating the inbreeding coefficient on the basis of runs of homozygosity, H_o_, and H_e_ in each experimental cattle breed (Table 1). We found that LFC and HNC displayed lower heterozygosity and higher inbreeding coefficients. Next, the *Ne* of HNC, LQC, and LFC populations 13 generations ago was estimated to be 63, 72, and 163, respectively. The *Ne* values of other breeds have been evaluated in previous studies (Xu et al., 2019b).

### Genetic Structure and Admixture Analyses

**Figure 1:**
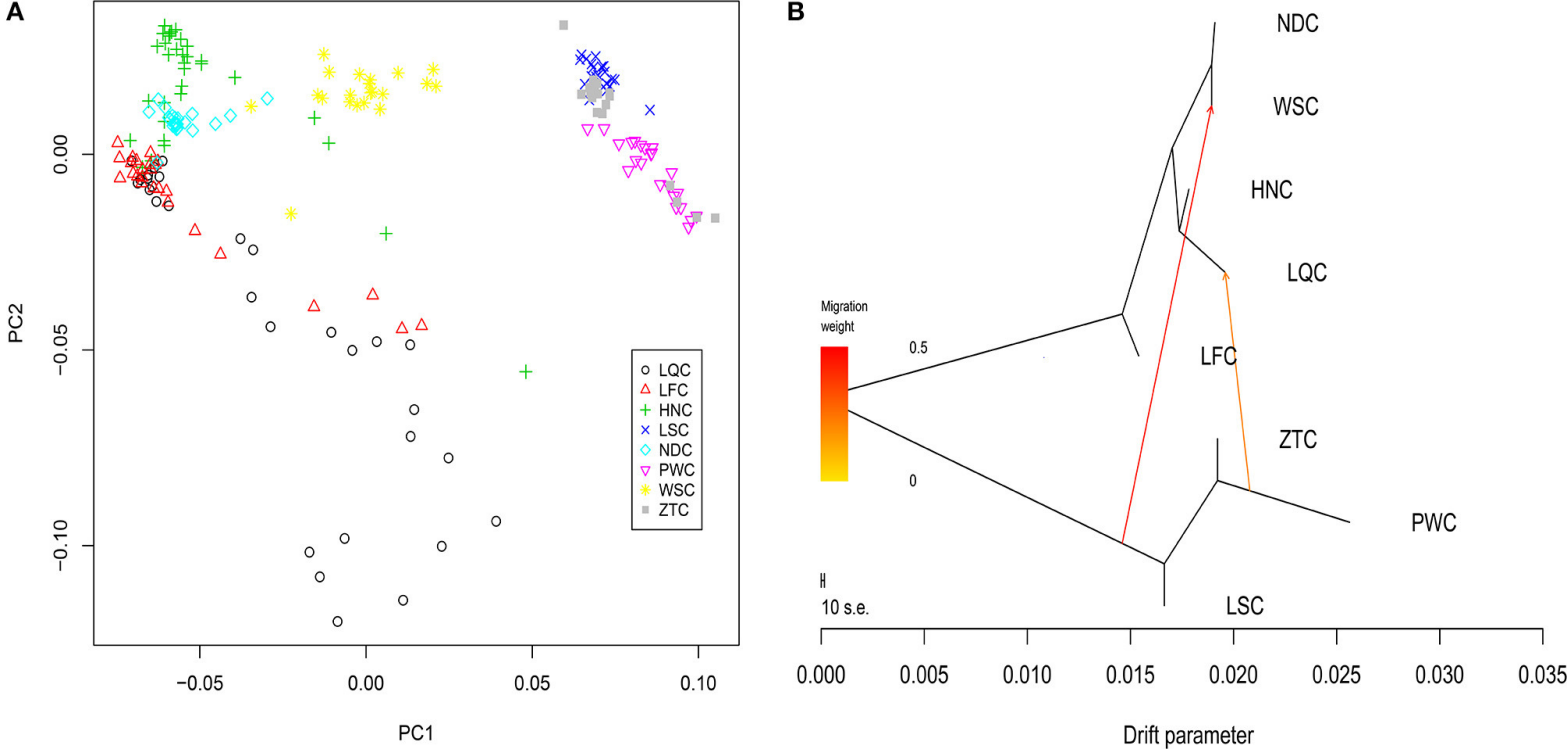
Population genetic analysis of eight experimental cattle breeds. **(A)** Multidimensional scaling (MDS) analysis results of 201 individuals. Individuals were plotted according to their coordinates on the first two components. **(B)** Maximum likelihood tree inferred from eight populations with two migration edges. The scale bar depicts 10 times the average standard error of the estimated entries in the sample covariance matrix.

Eight breeds were used to investigate the population genetic structure of and admixture in the local cattle breeds in southern China. Multidimensional scaling analysis showed a relatively well-defined population clustering among the eight breeds from different geographical areas. The result indicated that the first dimension separated ZTC, PWC, and LSC (from Yunnan and Sichuan provinces; primarily originated from taurine cattle) from HNC, NDC, LFC, and LQC (from Guangdong, Hainan, and Guangxi provinces; primarily originated from indicine cattle). The breed WSC was located in the middle of the two clusters, suggesting admixture from other breeds. In the second dimension, there was no obvious clustering, and LFC and LQC were scattered clustering well (Figure 1A).

Furthermore, we determined the degree of admixture in the eight cattle populations by performing admixture analysis using 11,455 LD-filtered SNPs with the number of clusters (K) varying from 2 to 10. At *K* = 2, we found that HNC, LFC, and LQC displayed a high degree of indicine ancestry. In contrast LSC, ZTC, and PWC exhibited large proportions of taurine content. Additionally, when *K* = 4 and 7, HNC, LFC, and LQC were admixed with several assumed lineages in a complicated fashion, while LSC, ZTC, and PWC show fewer lineages. At *K* = 9, we found that LFC contained a unique lineage (Supplementary Figure 1), which may indicate that LFC was distinguished from other breeds.

Next, a neighbor-joining tree was constructed (Purcell et al., 2007; Shimada and Nishida, 2017). We observed that animals from the same breed almost clustered together, and minor differences were observed in the internal branches within each breed (Supplementary Figure 2) (Cai et al., 2006, 2007). In addition, the clustering together of HNC and LQC was obvious, and these two breeds could not be clearly separated.

We further explored the admixture in these breeds by identifying the admixture events in the population using the maximum likelihood approaches in Treemix v1.13 software. The results of the admixture analysis were generally consistent with those of the STRUCTURE analysis. Moreover, we discovered some migration events and a certain degree of introgression between breeds. The first vector indicated gene flow from ZTC, PWC, and LSC (primarily taurine) to WSC (Figure 1B). The second vector suggested gene flow from ZTC, PWC, and LSC to LQC. In addition, the programs in Treemix v1.13 were used to estimate f3 and f4. The results suggested that the geographically proximate breeds (NDC|ZTC, LSC|NDC, and NDC|PWC) with the most extreme f3 scores (−71.75, −69.95, and −68.70, respectively) were the source of admixture for WSC. A similar pattern was observed for introgression into LQC from three populations (PWC, ZTC, and NDC) with extreme f3 scores (−38.72, −35.84, and −33.77, respectively). However, no significant results were obtained for the f4 test.

### Selection Signatures and Candidate Genes Under Selection

**Figure 2:**
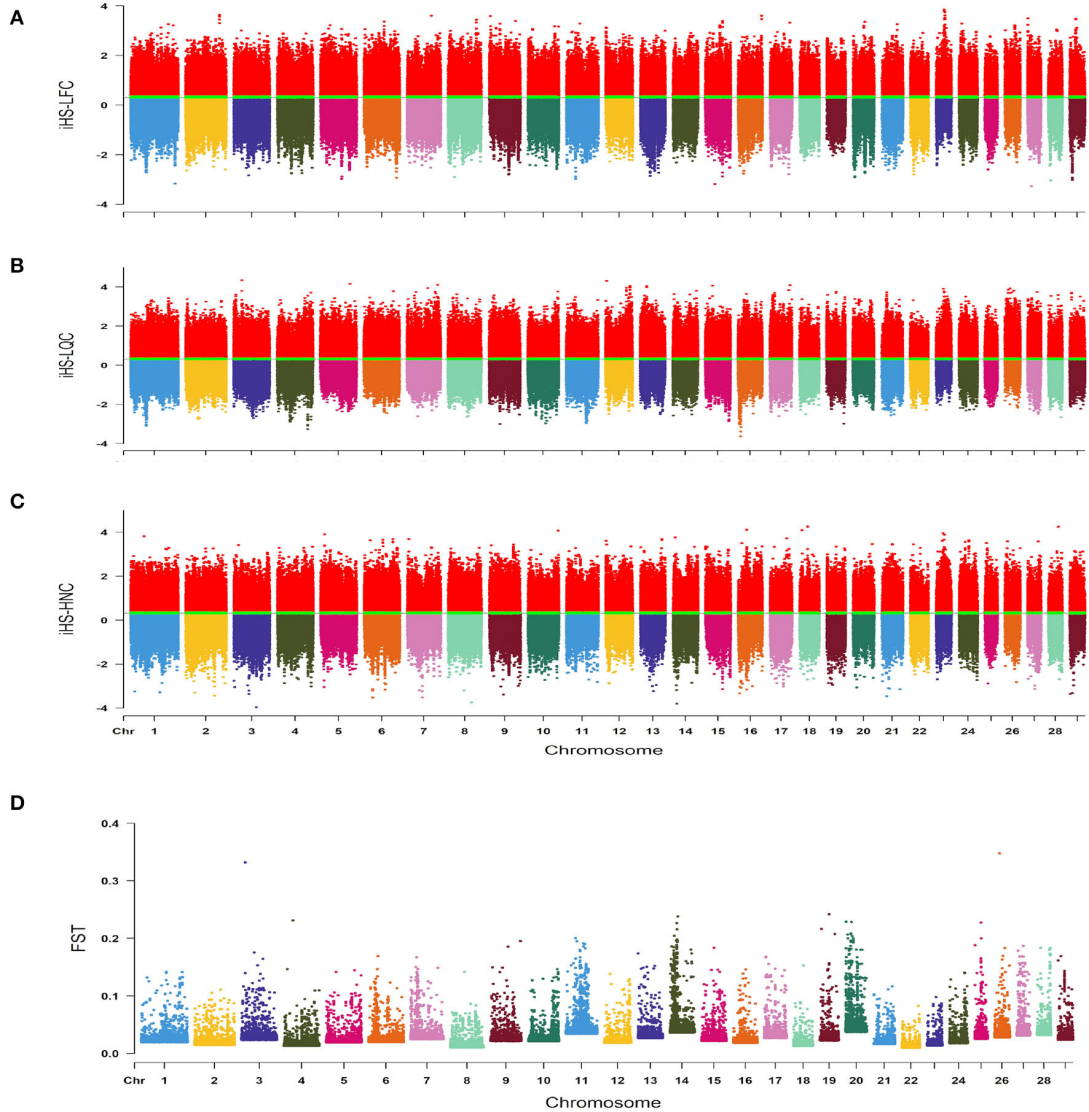
**(A)** Genome-wide distribution of integrated haplotype score (iHS) values estimated in Lufeng cattle (LFC). **(B)** Genome-wide distribution of iHS values estimated in Leiqiong cattle (LQC). **(C)** Genome-wide distribution of iHS values estimated in Hainan cattle (HNC). **(D)** Genome-wide distribution of F_ST_ values estimated in LFC, LQC, and HNC.

We explored the genomic regions under recent selection in southern Chinese local cattle breeds by estimating an iHS analysis of three representative cattle breeds (LFC, LQC, and HNC). The |iHS| values were estimated to visualize the distribution of selection signatures across autosomes (Figures 2A–C). In total, we identified 251, 270, and 256 candidate regions in LFC, LQC, and HNC, respectively. Based on the identified regions, we found 151, 185, and 155 genes under selection in LFC, LQC and HNC, respectively. The Rsb analysis estimated for three pairwise comparisons—LQC vs. LFC, LQC vs. HNC, and HNC vs. LFC—revealed 124, 132, and 119 candidate regions corresponding to 184, 174, and 146 candidate genes, respectively.

We investigated the potential genetic differences among the above three cattle breeds by estimating the F_*ST*_ values using BayeScan v2.1 (Figure 2D). After sorting the estimated F_*ST*_ values, a maximum of 76 SNPs sites were identified as outliers under selection; based on the *B. taurus* genome assembly (UMD 3.1/bosTau6), we identified 48 candidate genes located in the 50 kb regions upstream and downstream of the selected SNP sites (Supplementary Table 1). A total of 839 genes were detected; among these genes, there were 420, 354, and 43 unique genes for iHS, Rsb, and F_*ST*_ analysis, respectively (Supplementary Figure 3). Then, we performed the KEGG pathway and GO analysis based on the identified genes. In LFC, most genes were related to meat quality and immunity, whereas genes related to immunity were identified in LQC; however, genes with similar functions were not identified in NDC, KEGG pathway and GO analysis based on 48 candidate genes identified by F_*ST*_ analysis displayed these genes are related to immunity (hsa04514: Cell adhesion molecules). Detailed results of KEGG pathway and GO analysis were presented in Supplementary Figure 4.

## Discussion

We have previously investigated the population genetic characteristics of the eight cattle breeds in the current research (Xu et al., 2019a); however, genomic characteristics and selection signatures of the cattle from southern China were not fully explored. In this study, we conducted genetic structure, admixture, and phylogenetic analyses of local cattle breeds in southern China using a high-density SNP array. The results were generally consistent with the population history in cattle, which suggests that the local breeds in southern China are mainly indicine -derived populations (Yu et al., 1999). Analysis of the SNP data of the eight breeds in southern China suggested that HNC, LQC, LFC, and NDC primarily originated from indicine populations; LFC and HNC were clearly separated (Supplementary Figure 2), which indicated that these cattle had different genetic backgrounds. We also observed the gene flow from ZTC, PWC, and LSC to LQC, which could be explained that LQC had a more complicated genetic descent. Moreover, a higher inbreeding coefficient and a smaller effective group size also indicated a reduction in the number of local cattle breeds. Compared with previous studies that analyzed mitochondrial D-loop sequences, microsatellites and Y chromosomes in Chinese cattle, our analysis comprehensively refined the genetic relationship among local cattle breeds from southern China (Cai et al., 2006, 2007; Lai et al., 2006; Li et al., 2013). In farm animals, the genetic diversity of a local population is mainly caused by selection (natural and human-imposed) and non-selective forces (introgression and demographic events) (Wang et al., 2018). Identifying the genomic regions with selection signatures could provide valuable information about the effect of selection pressure for economically important and adaptive traits (Kemper et al., 2014; Zhao et al., 2015). This strategy could complement the current gene mapping approaches and further help to elucidate the genetic basis of complex traits (Andersson and Georges, 2004). We used three methods (intra-population (iHS), inter-population Rsb, and F_*ST*_) to identify candidate signatures of positive selection in HNC, LQC and LFC. A large number (815) of candidate genes were found; however, only 18 candidate genes were found by two of the methods, and only 1 candidate gene was found by three methods. The lower overlap between iHS and Rsb analyses may be explained by the reduced power of iHS to detect regions where alleles have almost reached fixation. Moreover, candidate genome regions identified by iHS might not be detected by Rsb if the favorable alleles/haplotypes were subjected to selection in the reference populations (Bahbahani et al., 2015). Poor overlap among Rsb, iHS, and F_*ST*_ analysis might result from the selection of timescale. Studies showed that the iHS and Rsb methods are more suitable for detecting the recent selection signal of the genome (age of selection <1,200), while the F_*ST*_ method is suitable for the ancient selection signal (age of selection <3,000) (Utsunomiya et al., 2013). We attempted to detect both recent selection and ancient selection signatures among the breeds HNC, LQC, and LFC using a high-density genotype array (Qanbari et al., 2010). Using the iHS approach, we detected 151, 185, and 155 genes under selection in the LFC, LQC, and HNC breeds, respectively, and these genes were found to be associated with meat quality, fatty acids, immunity, and homeostasis.

We totally detected 41 overlapped candidate genes compared with the previous studies (Chen et al., 2018; Xu et al., 2019a). The recessive mutation in *RSPO2* gene could cause tetra-amelia syndrome, which is characterized by lung aplasia and the total absence of the four limbs (Szenker-Ravi et al., 2018). *COL8A1* gene promotes the proliferation of smooth muscle cells (Li et al., 2018). *BMP10* plays important roles in angiogenesis (Lei et al., 2016). *ALCAM* gene is reported to be involved in T-cell activation, development, inflammation and transendothelial migration of neutrophils (Weidle et al., 2010; May et al., 2019). In addition, we identified some candidate genes related to important economic traits. Of them, three genes (*CLSTN2, DPYD*, and *CHSY3*) are found to be related to meat quality and fatty acids in LFC cattle. *CLSTN2* (calmodulin 2) plays an important role in promoting adipocyte proliferation in visceral adipose tissue and subcutaneous fat and is associated with mammalian obesity (Santana et al., 2015). Dihydropyrimidine dehydrogenase (*DPYD*) could increase the marbling fat (Lim et al., 2014). The *CHSY3* plays an important role in regulating meat tenderness (Leal-Gutierrez et al., 2019). In general, these genes might explain the superior meat quality of the local cattle in southern China.

Meanwhile, some candidate genes for adaptive traits were also found in our study. We identified four genes (*BCAR3, PRNP, TRAPPC9*, and *DNAJC2*) linked to immunity and homeostasis in LFC cattle. *BCAR3, PRNP*, and *TRAPPC9* have been previously reported to potentially regulate specific diseases. *BCAR3* is related to the entry of *Mycobacterium avium* ssp. *paratuberculosis* into the host cell or the immune response initiated in response to a *Mycobacterium avium* ssp. *paratuberculosis* infection (Kiser et al., 2018). Although polymorphisms in the *PRNP* promoter do not confer absolute disease resistance, *PRNP* regulates the susceptibility to bovine spongiform encephalopathy (Haase et al., 2007). *TRAPPC9* is a candidate gene for the regulation of susceptibility to mastitis in Holsteins (Wang et al., 2015). Additionally, genes (*TMSB4, TGM3*, and *LTF*) with similar functions are also found in LQC cattle. *TMSB4* plays an important role in the development of the immune system; it encodes thymosin b4, which is a hormone mainly involved in functions such as endothelial cell differentiation, angiogenesis, inflammation, wound healing, apoptosis, and tumorigenesis (Salhaba et al., 2010). A previous study suggested that *TGM3* plays a key role in host response to tick infestation, which may explain the strong resistance of LQC to insects in the wild (Taye et al., 2018). More remarkably, *LTF* encodes a multifunctional protein with antibacterial properties; it is an important component of innate immunity and is active against many species of gram-negative and gram-positive bacteria, enveloped and non-enveloped viruses, and various types of fungi and parasites (Wojdak-Maksymiec et al., 2013). *DNAJC2* is involved in homeostasis; it could drive the cellular response to heat stress; this gene is also related to the ability of LFC cattle to better adapt to the natural hot and humid climate of the region (Verma et al., 2018).

Using the BayeScan v2.1 software, we identified 76 loci under selection, and a total of 48 genes under selection based on the identified regions. We found that many genes were related to immunity and adaptability based on the KEGG analysis. In the present study, three genes (*CDH5, NRXN1*, and *NEGR1*) were found to be enriched in the cell adhesion molecular pathway, which is related to immunity and disease. *CDH5* promotes the growth of endothelial cells and maintains the normal function of endothelial cells. CDH5 is one of the main components of endothelial adhesion and connection, which maintains the integrity of the endothelial barrier and controls the migration of leukocytes during injury and infection (Chamorro-Jorganes et al., 2016; Gu et al., 2017). Both *NLGN1* and *NEGR1* are involved in mammalian nerve development, and they may induce neurological diseases. *NLGN1* encodes the synaptic protein Neuroligin 1, which is involved in synapse formation and remodeling (Samarelli et al., 2014). Studies have shown that *NRXN1-*α knockout mice exhibit social behavior disorders and increased autism-like symptoms such as aggressive behavior (Trezza et al., 2013), whereas variations in the *NEGR1* gene regulatory region are associated with major depressive disorder (Wang et al., 2020).

However, it remains difficult to determine the relationship between the genes identified in the present study and the adaptive characteristics of the local cattle in southern China; the functional study and validation of causal variants should be performed in the future. Generally, our results provide important insights into the genomic selection signature in local cattle breeds in southern China. We identified a series of breed-specific candidate genes under selection, which are involved in immune response and meat quality, suggesting that these genes have been under differential selection pressure in various environmental conditions.

## Data Availability Statement

This article contains previously unpublished data. The name of the repository and accession number(s) are not available.

## Author Contributions

YLiu and LX drafted the manuscript and performed the experiments. YLiu and DL collected the blood samples. YLiu, GZ, and LY performed data analysis. LX, DL, and YLi conceived the study and participated in its design and coordination and helped to draft the manuscript. All authors have read and approved the final manuscript.

## Conflict of Interest

The authors declare that the research was conducted in the absence of any commercial or financial relationships that could be construed as a potential conflict of interest.
