# Supplementary material for: Discovery of Genomic Characteristics and Selection Signatures in Southern Chinese Local Cattle
Source: Front Genet. 2020 Dec 18;11:533052. doi: 10.3389/fgene.2020.533052 (PMC7775540; doi:10.3389/fgene.2020.533052)
Supplement: Supplementary file 1 [file Table_3.DOCX]

article

## Supplementary Figures


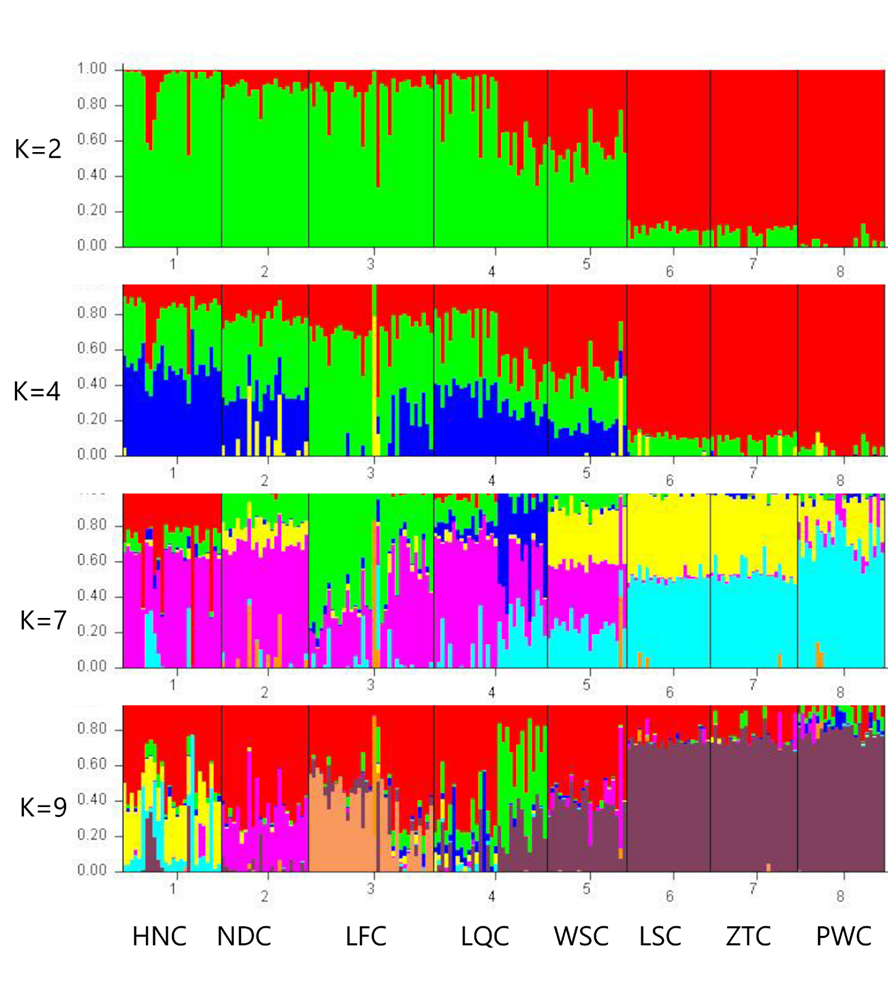


**Supplementary Figure S1.** Clustering of 201 individuals based on linkage disequilibrium-filtered single nucleotide polymorphisms (SNPs) with the number of clusters (K) ranging from 2 to 9. Individuals are shown as a thin vertical line colored in proportion to their estimated ancestry.


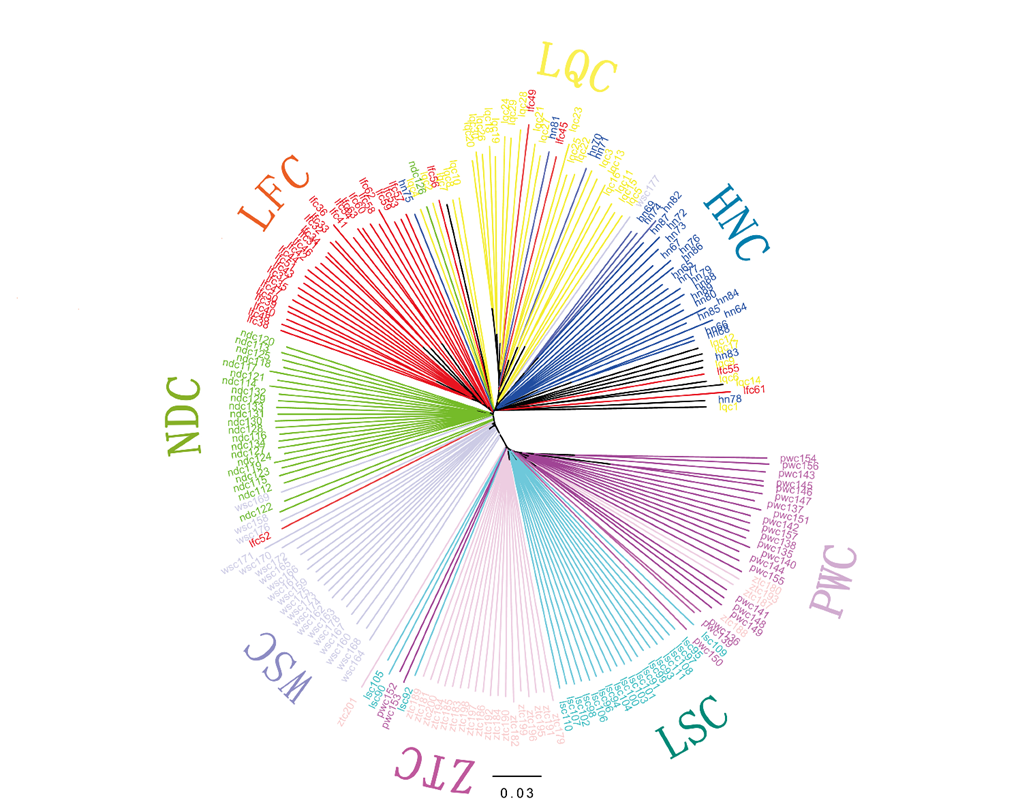


**Supplementary Figure S2.** Neighbor-joining tree of the 201 individuals from eight breeds of Chinese cattle. The tree was constructed using the allele sharing distance averaged over 568,129 SNPs. Edges are colored according to the individuals’ breed of origin.


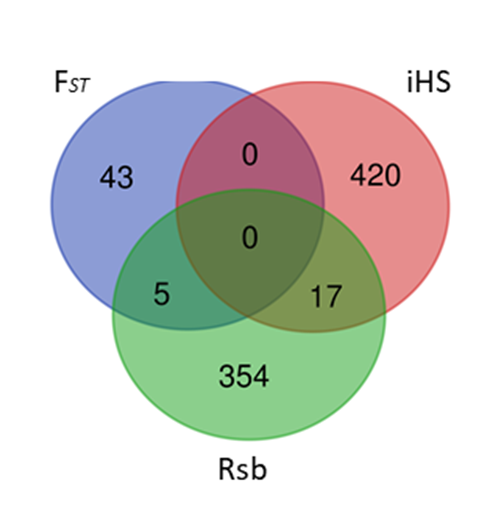


**Supplementary Figure S3.** Venn diagram illustrating the candidate genes for the F*_ST_*, iHS and Rsb analysis.


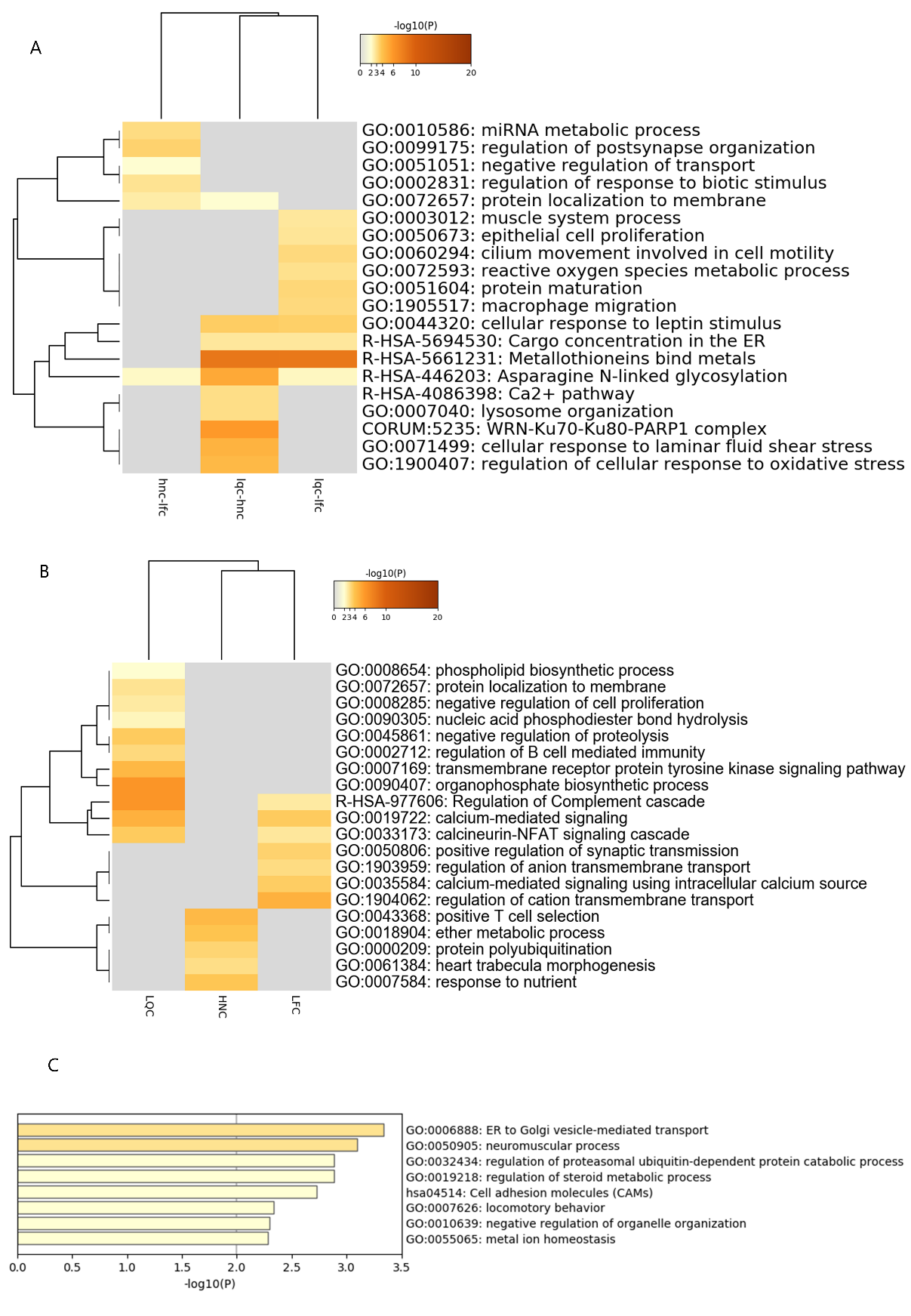


**Supplementary Figure S4.** (A)The result of KEGG pathway and GO analysis based on candidate genes determined by Rsb analysis. (B) The result of KEGG pathway and GO analysis based on candidate genes determined by iHS analysis. (C) The result of KEGG pathway and GO analysis based on candidate genes determined by F*_ST_* analysis.
